# Online User Information Sharing and Government Pandemic Prevention and Control Strategies-Based on Evolutionary Game Model

**DOI:** 10.3389/fpubh.2021.747239

**Published:** 2021-11-15

**Authors:** Yao Xiao, Wanting Xu, Shouzhen Zeng, Qiao Peng

**Affiliations:** ^1^Center for Innovation and Development Studies, Beijing Normal University, Zhuhai, China; ^2^Economics and Resource Management, Beijing Normal University, Beijing, China; ^3^School of Business, Ningbo University, Ningbo, China; ^4^School of Statistics, Tianjin University of Finance and Economics, Tianjin, China

**Keywords:** online users, government, information sharing, privacy protection, COVID-19 prevention and control

## Abstract

**Background:** The sharing and utilization of online users' information has become an important resource for governments to manage COVID-19; however, it also involves the risk of leakage of users' personal information. Online users' sharing decisions regarding personal information and the government's COVID-19 prevention and control decisions influence each other and jointly determine the efficiency of COVID-19 control and prevention.

**Method:** Using the evolutionary game models, this paper examines the behavioral patterns of online users and governments with regard to the sharing and disclosure of COVID-19 information for its prevention and control.

**Results:** This paper deduce the reasons and solutions underlying the contradiction between the privacy risks faced by online users in sharing information and COVID-19 prevention and control efforts. The inconsistency between individual and collective rationality is the root cause of the inefficiency of COVID-19 prevention and control.

**Conclusions:** The reconciliation of privacy protection with COVID-19 prevention and control efficiency can be achieved by providing guidance and incentives to modulate internet users' behavioral expectations.

## Introduction

Information sharing and utilization by online users has become an important resource for governments to manage the spread of COVID-19. In 2020, the rapid proliferation of the virus severely tested the national governance capacity of global countries. Owing to the continuous development and penetration of the Internet in recent years, big data, cloud computing, artificial intelligence, and other scientific technologies are being widely used in all aspects of COVID-19 prevention and control; to this end, extensive collection, processing, and investigation of online users' personal information are currently underway. Governments in all countries have attached great importance to the collection, sharing, and disclosure of COVID-19 information. Personal information regarding confirmed patients, suspected patients, and close contacts is collected, collated, and disseminated to society through appropriate channels, to mitigate public panic and simultaneously remind the public to be actively alert and take protective measures. These initiatives have played an important role in enhancing the timeliness and accuracy of the execution of COVID-19 prevention and control measures. Although the sharing and utilization of COVID-19 data can certainly contribute to combating the pandemic, the continuous release of such information by governments increases the risk of personal information being leaked (1). As a result of this illegal disclosure of personal information, the private lives of many citizens especially the patients have been severely affected, with incidents of individuals being bombarded with text messages, abusive phone calls, and even personal attacks reported. With the deepening of the pandemic, more and more cases of discrimination against citizens especially patients have been reported.

Therefore, there is a trade-off between the precise and effective prevention and control of COVID-19 (by governments) and the protection of public personal information. The government may collect personal information related to COVID-19 through apps online. If online users take the initiative to download the app and fill in or share personal details, the government can collect more accurate information about the users and manage the spread of COVID-19 more effectively and precisely. However, some online users may not voluntarily provide personal information to the government, especially those who realize that the government may disclose their personal information. Although the government will offer anonymization of user information, the possibility of data breaches is still high especially in the digital era. Therefore, some online users do not provide personal information to the government, which, in turn, affects the effectiveness of COVID-19 prevention and control. Furthermore, the effectiveness of COVID-19 management may affect users' willingness to share personal information. Thus, the online users also face a trade-off between protecting their interests (sharing personal information with the government) and the effective management of COVID-19 (which ultimately safeguards their health). The benefits and decisions of online users and governments are interlinked, and the mutual decisions also affect the effectiveness of COVID-19 prevention and control.

The nature of the game relationship between online users and the government renders the game theory model especially the evolutionary game theory models effective for studying the decisions of multiple interested parties. Evolutionary game theory abandons the assumption of perfect rationality in classical game theory, and replaces it with the assumption of limited rationality (2). The assumption is that the participants are not rational individuals with infinite reasoning abilities. They cannot precisely calculate the Nash equilibrium strategy and make the corresponding choice, like individuals in traditional games (3); however, they can learn and adjust their own strategies and gradually converge to a stable Nash equilibrium strategy according to the results of each game in the process of continuous repetition (4). Based on the idea of evolution in biology, the evolutionary game theory adopts the strategy type that participants can choose as the gene type, and expresses “fitness” through the income obtained by participants on selecting a certain strategy. Individuals with limited rationality determine the probability of a strategy being selected based on the principle of pursuing the maximization of interests to replace natural selection in biological evolution: the greater the benefit of a strategy, the greater the probability that the strategy will be selected again; that is, corresponding to the “heredity” in biology, the participants will constantly adjust their strategy according to the size of the benefit, until all participants no longer adjust their strategies. Therefore, by constructing an evolutionary game model of the government's COVID-19 prevention and control strategies and the sharing of personal information by online users, the complexity and uncertainty in the decision problem of COVID-19 prevention and control and the sharing of personal information by online users can be accurately portrayed. The evolutionary game model can also provide a good analytical framework for studying the problem of COVID-19 management and online user privacy protection.

## Literature Analysis

The issue of COVID-19 prevention and control and the protection of personal information of online users has become a widely discussed topic in current academic research. Research has been conducted in three main areas.

(1) Precise digital management of COVID-19 from the perspective of anonymization technologies for online user personal information: Zhiwei et al. (5), Cheng and Hao (6), and Elkhodr et al. (7) all argue that privacy protection issues have become a major obstacle to the adoption of COVID-19 tracing apps and big data technologies which aimed at curbing the spread of the pandemic. Targeted improvement of data anonymization techniques in these apps and big data technologies can achieve precise COVID-19 management while protecting personal privacy information as well. Sharma et al. (8) investigated the use and permissions of user personal information on 50 apps related to the COVID-19 information collection and determined their impact on related user privacy protection laws. Wu et al. (9) and Gerke et al. (10) assessed the levels of security and privacy protection on current mainstream COVID-19 tracing apps by using various methods. (2) Privacy protection in the prevention and control of COVID-19 from the perspective of reconstructing the data collection rules or privacy protection laws: Vitak and Zimmer (11) advocated the construction of an entire personal information protection framework from privacy protection-oriented perspectives. They insisted that this framework be applied to data collection, processing, and other uses in the context of COVID-19 prevention and control. The framework is expected to form a benign ecology of legal personal information protection and ensure the responsible use of personal information. Newlands et al. (12) investigated digital surveillance technologies implemented during COVID-19 and their impact on personal information privacy through case studies. They explored the ways to accelerate the creation of privacy assessment standards to establish regulatory technologies and laws that can effectively mitigate privacy risks. Azad et al. (13) conducted an analysis of a large set of smartphone applications designed to curb the spread of COVID-19. They argued that user privacy can be ensured by regulating the types of licenses, permissions, and security regulations for data application and analysis across applications, thus allowing people to return to normal life. (3) Analysis of online users' preferences and choices of COVID-19 information tracing apps and behaviors such as information sharing: Sharma et al. (8) assessed the privacy control status of COVID-19 tracing apps through questionnaires and further explored the online users' preferences for the apps. They found that the degree of privacy protection was a determining factor for online users in choosing a COVID-19 tracing app. Meanwhile, Klar and Lanzerath (14) argued that the deployment of COVID-19 tracing apps that are effective in preventing the spread of the virus and it greatly benefits society; however, the fear of privacy breaches leads to reluctance in acceptance by many users, making it difficult to effectively manage the virus. He advocated an ethical approach to micro-force public acceptance of apps. Hohman et al. (15) studied the selection behavior of COVID-19 tracing apps of specific populations and argued that a combination of effective communication strategies and maintaining appropriate social distance could facilitate the popularity of the apps and thus, improve the prevention and control of the pandemic. Wottrich et al. (16) drew on Roger's Protection Motivation Theory (PMT) to analyze the preferences and choices of 1,593 Western European COVID-19 tracing app users and found that users' self-efficacy, vulnerability, and level of privacy concern influence their choice of application, and frequency and depth of use. Based on privacy calculation theory, Yue (17) studied the privacy disclosure behaviors of web or app users, and found that users usually weigh privacy disclosure risks and benefits when making decisions on whether to disclose private information or not. The results of their calculations and weightage revealed different levels of privacy concerns, which in turn played a key role in users' privacy disclosure behavior. Other scholars (18–22) have argued that, as a type of user privacy behavior, internet users' privacy disclosure behavior is closely related to their privacy concerns; thus, all these studies conclude that there is a high correlation between information privacy concerns and personal information disclosure behavior. However, there is no agreement on whether this correlation is positive or negative. Other scholars (23, 24) have denied that privacy concerns have an impact on users' personal information disclosure behavior; that is, the privacy paradox, where users' privacy concerns are inconsistent with their personal information disclosure behaviors or are irrelevant. Users express concern about their data leakage on the one hand, but actively disclose a large amount of personal information on the other hand (25, 26). Some scholars have used rational choice models in economics to study the personal information disclosure behaviors of online users. Social benefits, personalized services, and privacy benefits are the main factors determining whether users share their personal information through apps or other Internet platforms (27–34). Therefore, rewards and privacy policies can all influence online users' privacy-sharing decisions (21, 35, 36). However, whether online users remain completely rational in their decision-making processes remains debatable (37). There is also a considerable amount of research examining the personal information sharing behavior of online users from a psychological perspective, arguing that the users' privacy sensitivity, desire for privacy protection, personality, and emotions can have a significant impact on their personal information sharing behavior (38–42).

It is sure that the government can help control the spread of COVID-19 through the collection and use of related personal information from online users, the privacy of online users can also be protected to some extent through improved data anonymization techniques. However, there remains a paradox that the greater the degree of anonymity of user information, the lower the effectiveness of COVID-19 management. The government not only collects user information online for precise resource allocation and to take appropriate measures to manage COVID-19, but also responds to the pandemic by disclosing the collected information to the public to remind them to proactively plan their activities, especially travel. The disclosure of anonymized online user information protects user privacy but weakens the management of COVID-19. Therefore, there is a trade-off between privacy protection and disclosure of user information for COVID-19 management, which requires further investigation. Most current studies on privacy protection and the management of COVID-19 mainly focus on the study of online users' privacy information sharing behavior or view online users as rational or irrational individuals, from economic, psychological, and sociological perspectives. Few scholars consider the government's behaviors and decisions regarding privacy protection and COVID-19 management. In particular, under the premise that the behaviors and decisions of the government and the public interact with each other, there are almost no scholars who have included both aspects—COVID-19 management by the government and user privacy protection—in the same framework to study the final results of their mutual influence and the corresponding social efficiency. Therefore, this study makes the following marginal contributions to the existing literature: First, we adopt a game theory model to include both the government and online users into the analytical framework. The information sharing decisions of online users, as well as the government's prevention and control decisions of COVID-19 are analyzed, as are the equilibrium outcomes of their mutual games and the corresponding social efficiency. Second, drawing on the assumption of irrational individuals in psychology, we adopt evolutionary game theory models to analyze the learning and adjustment process of government's prevention and control decisions, and online users' information sharing decisions.

## Research Design

### Model Building

In this section, a framework of evolutionary game theory is used to construct an evolutionary game model that involves online users and the government to analyze the interactions between online users' decisions on information sharing and the government's decisions on pandemic prevention and control. Concerning the behavior patterns of the participants, the government and online users, the following assumptions are made.

In choosing whether to share personal information with the government for the sake of pandemic control, online users seek to maximize their self-interest. When the pandemic is under control, the personal benefits to users increases. However, the sharing of personal information also increases risks of privacy breach.

The government chooses whether to disclose personal information collected from the users, thereby helping the general public deal with the pandemic by arranging their daily lives and travel plans rationally or adopting corresponding measures. Nevertheless, even if the users do not share personal information, the government will collect their information through other means, albeit with lower information precision. The government is an entity that maximizes the public interest in society, by handling the pandemic, maintaining social stability, and ensuring the stability of the economy. However, as the government discloses user information related to the pandemic, it has to bear the financial and time costs incurred by legal disputes concerning matters such as privacy leakage.

The factors related to the process of pandemic prevention, control, and sharing of privacy are quantified to analyze the behavior decisions of online users and the government as follows: When online users do not share personal information with the government, the government can still collect, with a certain accuracy, users' personal information through some channels; when the government also decides not to disclose the information it collected on the epidemic to the public, that is, in the state of (not sharing, not disclosing), the total value of the information to the society is *V*. When online users share personal information with the government, the government obtains more accurate and thus more valuable user information, so that the total value of the information generated to the society is α*V*, where α> 1 indicating that the value generated by the personal information shared by the users is higher compared to the government's own collection. The more accurate the personal information shared by the users, the greater the value produced to the society, that is, α would be greater. Therefore, in the state of (sharing, not disclosing), the total value of the information to the whole society is α*V*, because when the government discloses the collected user information to the general public, the public can plan their personal life and travel in a way that is more conducive to epidemic control. Suppose the extra value created by the government disclosing the collected user information is *L*. However, the disclosure of user information also leads to social cost, which contains two parts borne by the user and the government respectively. First, users' interest is hurt by the government's disclosure of their private information. Second, the government, after being blamed by the users for the disclosure, not only suffers a loss of its image but has to spend time and money in dealing with various privacy disputes. Therefore, in the state of (no sharing, disclosure), the total social value generated by the information is (*V*+*L*) −*C*. When the government collects the epidemic information shared by online users and discloses it, the total social value generated is α(*V*+*L*), but as the information thus shared and disclosed is more precise, namely, more private, the disclosure leads to higher social costs γC, where γ > 1, and the more precise the information shared by users, the greater the social costs, that is, would be greater. For online users, this means a greater loss to personal interests because the shared information can be misused by others or certain companies, whereas the government is more likely to not only suffer more accusations and criticisms but also get involved in more privacy disputes. Therefore, in the (share, disclose) state, the total social value generated by the information is α(*V*+*L*)−γC. To simplify the model, this paper further assumes that the total social value generated by the epidemic information is distributed between online users and the government according to an unchanged ratio β/(1-β).

Therefore, based on the above assumptions, the payoff matrix of the game of the government and online users can be calculated, as shown in Table 1. This study further assumes that the government and users are bounded rationality agents, who constantly adjust their strategies to maximize self-interest based on the information they obtain. Therefore, there are uncertainties in their decisions, that change continuously according to the other players' strategies. It is assumed that an online user chooses to share personal information with the government with a probability denoted by, and constantly adjusts the probability to maximize their self-interest. It is also assumed that the government chooses to disclose user information to society with a probability denoted by *y*, thereby preventing and controlling the pandemic more effectively and constantly adjusting the probability to maximize its self-interest. The replicator equation is employed to adjust the shifts in the government's strategies and online users, and is expressed as follows (43).


$$\begin{array}{l} F\left(x_{v}\right)=\frac{dx_{v}}{dt}=x_{v}\left[E\left(x_{v}\right)-\bar{E}\right] \end{array}$$

where *x*_*v*_ denotes the probability of a player adopting a strategy *v*, *E*(*x*_*v*_) denotes the player's expected payoff in adopting the strategy *v*, $\bar{E}$ denotes the average payoff gained as the player adopts all possible strategies.

**Table 1:** Payoff matrix of the game of the government and online users in information sharing and disclosure.

		**The government**	
		**To disclose**	**Not to disclose**
Online users	To share	αβ(*V*+*L*)−γ*βC*, (1−β)α(*V*+*L*)−(1−β)γ*C*	α*βV*, α(1−β)*V*
	Not to share	β(*V*+*L*)−β*C*, (1−β)(*V*+*L*)−(1−β)*C*	β*V*, (1−β)*V*

### Model Solving

Using the payoff matrix of the game of the government and online users, together with the replicator equation, a replicator equation that describes the strategy adjustments of online users and the government can be obtained.

(1) Set *U*_*p*, 0_ as the expected payoff when an online user chooses to share personal information, *U*_*p*, 1_ as the expected payoff when the online user chooses not to share personal information, and *U*_*p*_ as the average payoff gained by the online user.

Among them,


(1) $$\begin{array}{r} U_{p,0}=y\left[\alpha \beta \left(V+L\right)-\gamma \beta C\right]+\left(1-y\right)\alpha \beta V \end{array}$$

(2) $$\begin{array}{r} U_{p,1}=y\left[\beta \left(V+L\right)-\beta C\right]+\left(1-y\right)\beta V \end{array}$$

(3) $$\begin{array}{r} U_{p}=xU_{p,0}+\left(1-x\right)U_{p,1} \end{array}$$

Thus, the replicator equation that describes the strategy adjustment of online users is as follows:


(4) $$\begin{array}{l} \frac{dx}{dt}=x(U_{p,0}-U_{p})=x(1-x)\{[(\alpha -1)\beta L-(\gamma -1)\beta C]y\\ \;+(\alpha -1)\beta V\} \end{array}$$

When the strategy adjustment of the online user tends to stabilize, that is, when $\frac{dx}{dt}=0$,


(5) $$\begin{array}{r} x_{1}^{*}=0,x_{2}^{*}=1,y_{3}^{*}=b=\frac{\left(\alpha -1\right)\beta V}{\left(\alpha -1\right)\beta L-\left(\gamma -1\right)\beta C} \end{array}$$

(2) Set *U*_*g*, 0_ as the expected payoff when the government chooses to disclose the personal information of the online user, *U*_*g*, 1_ as the expected payoff when the government chooses not to disclose the personal information of the online user, and *U*_*g*_ as the average payoff gained by the government.


(6) $$\begin{array}{l} U_{g,0}=x[(1-\beta )\alpha (V+L)-(1-\beta )\gamma C]+(1-x)[(1-\beta )\\ \;(V+L)-(1-\beta )C] \end{array}$$

(7) $$\begin{array}{rcl} U_{g,1} & = & x\left(1-\beta \right)\alpha V+\left(1-x\right)\left[\left(1-\beta \right)V\right] \end{array}$$

Similarly, the replicator equation that describes the government's strategy adjustment is as follows:


(8) $$\begin{array}{l} \frac{dy}{dt}=y(U_{g,0}-U_{g})=y(1-y)\{x(1-\beta )[(\alpha -1)L-(\gamma -1)C]\\ \;+(1-\beta )(L-C)\} \end{array}$$

When the strategy adjustment of the government tends to be stable, that is, when $\frac{dy}{dt}=0$,


(9) $$\begin{array}{r} y_{1}^{*}=0,y_{2}^{*}=1,x_{3}^{*}=a=\frac{L-C}{\left(\alpha -1\right)L-\left(\gamma -1\right)C} \end{array}$$

When $\frac{dx}{dt}=0$ and $\frac{dy}{dt}=0$, four equilibrium points exist in the evolutionary game of the government and online users regarding information disclosure and pandemic prevention and control; the points are (0, 0), (0, 1), (1, 0), (1, 1), and (*a, b*) respectively.

### Stability Analysis of the Equilibrium Solution

According to the analysis in the previous section, the dynamic adjustment process of online user and government strategies can be represented by the following set of differential equations that form a two-dimensional dynamical system with five equilibrium points: (0, 0), (0, 1), (1, 0), (1, 1), and (*a, b*).


(10) $$\begin{array}{l} F(x,y)=\frac{dx}{dt}=x(1-x)\{[(\alpha -1)\beta L-(\gamma -1)\beta C]y\\ \;+(\alpha -1)\beta V\} \end{array}$$

(11) $$\begin{array}{l} G(x,y)=\frac{dy}{dt}=y(1-y)\{x(1-\beta )[(\alpha -1)L-(\gamma -1)C\\ \;+(1-\beta )(L-C)\} \end{array}$$

To determine the stability of the equilibrium points in a two-dimensional non-linear dynamical system, a first-order Taylor expansion must be performed at each equilibrium point (x^*^, y^*^) for *F*(*x, y*) and *G*(*x, y*) through a linear approximation, that is,


(12) $$\begin{array}{rcl} \frac{dx}{dt} & = & F_{x}\left(x^{*},y^{*}\right)\left(x-x^{*}\right)+F_{y}\left(x^{*},y^{*}\right)\left(y-y^{*}\right) \end{array}$$

(13) $$\begin{array}{rcl} \frac{dx}{dt} & = & G_{x}\left(x^{*},y^{*}\right)\left(x-x^{*}\right)+G_{y}\left(x^{*},y^{*}\right)\left(y-y^{*}\right). \end{array}$$

Then, the coefficient matrix of this two-dimensional dynamical system (the Jacobi matrix) is denoted by


(14) $$\begin{array}{r} J=\left[\begin{array}{cc} \frac{\partial F\left(x,y\right)}{\partial x} & \frac{\partial F\left(x,y\right)}{\partial y}\\ \frac{\partial G\left(x,y\right)}{\partial x} & \frac{\partial G\left(x,y\right)}{\partial y} \end{array}\right]=\left[\begin{array}{cc} c_{11} & c_{12}\\ c_{21} & c_{22} \end{array}\right], \end{array}$$

where


(15) $$\begin{array}{rcl} c_{11} & = & \left(1-2x\right)\left\{\left[\left(\alpha -1\right)\beta L-\left(\gamma -1\right)\beta C\right]y+\left(\alpha -1\right)\beta V\right\} \end{array}$$

(16) $$\begin{array}{rcl} c_{12} & = & x\left(1-x\right)\left[\left(\alpha -1\right)\beta L-\left(\gamma -1\right)\beta C\right] \end{array}$$

(17) $$\begin{array}{rcl} c_{21} & = & y\left(1-y\right)\left(1-\beta \right)\left[\left(\alpha -1\right)L-\left(\gamma -1\right)C\right] \end{array}$$

(18) $$\begin{array}{l} c_{22}=(1-2y)\{x(1-\beta )[(\alpha -1)L-(\gamma -1)C]\\ \;+(1-\beta )(L-C)\} \end{array}$$

The stability of the equilibrium point is determined by the eigenroots of the Jacobi matrix *J*. We denote the eigenroots by λ_1_ and λ_2_, which are determined by the characteristic equation of the Jacobi matrix *J*.


(19) $$\begin{array}{r} \det\left(J-\lambda I\right)=0 \end{array}$$

By including the elements of the Jacobi matrix *J* in Equation (19), the above characteristic equation can be expressed as


(20) $$\begin{array}{r} \lambda^{2}-\left(c_{11}+c_{22}\right)\lambda +c_{11}c_{22}-c_{12}c_{21}=0 \end{array}$$

The solution of the above two-dimensional dynamical system in the vicinity of the equilibrium point (x^*^, y^*^)can be expressed as


(21) $$\begin{array}{r} \left(\begin{array}{c} x\left(t\right)\\ y\left(t\right) \end{array}\right)=\left(\begin{array}{c} b_{11}\\ b_{21} \end{array}\right)e^{\lambda_{1}t}+\left(\begin{array}{c} b_{12}\\ b_{22} \end{array}\right)e^{\lambda_{2}t}+\left(\begin{array}{c} x^{*}\\ y^{*} \end{array}\right) \end{array}$$

In the game between the government and online users, only the stable equilibrium points are considered, while the unstable ones are difficult to retain. Therefore, this study only focuses on stable equilibrium points while excluding the unstable points, such as saddle points. According to Equation (21), the two-dimensional dynamical system converges to the stable equilibrium point (x^*^, y^*^) only when λ_1_ < 0 and λ_2_ < 0 (In the application setting of evolutionary games, imaginary characteristic roots are almost unlikely to appear, so only the real characteristic root case is discussed in this paper).

From the characteristic Equation (20), it follows that


(22) $$\begin{array}{rcl} \lambda_{1}+\lambda_{2} & = & c_{11}+c_{22} \end{array}$$

(23) $$\begin{array}{rcl} \lambda_{1}\lambda_{2} & = & c_{11}c_{22}-c_{12}c_{21} \end{array}$$

Therefore, the necessary and sufficient conditions to ensure that λ_1_ < 0 and λ_2_ < 0 are


(24) $$\begin{array}{r} c_{11}+c_{22}<0 \end{array}$$

(25) $$\begin{array}{r} c_{11}c_{22}-c_{12}c_{21}<0 \end{array}$$

According to the Jacobian matrix of the replicator equation, Table 2 can be obtained:

**Table 2:** Analysis of local equilibrium points.

**Equilibrium point**	***c*_11_**	***c*_12_**	***c*_21_**	***c*_22_**
(0,0)	(α−1)β*V*	0	0	(1−β)(*L*−*C*)
(0,1)	(α−1)β(*V*+*L*)−(γ−1)β*C*	0	0	−(1−β)(*L*−*C*)
(1,0)	−(α−1)β* V*	0	0	−(1−β)(α*L*−γ*C*)
(1,1)	−(α−1)β(*V*+*L*)+ (γ−1)β*C*	0	0	−(1−β)(α*L*−γ*C*)
(a,b)	0	$c_{12}^{*}$	$c_{21}^{*}$	0

According to the stability conditions of the equilibrium solution in the evolutionary game, we determine the stability condition of each equilibrium point:

(1) When (α−1)β(*V*+*L*) < (γ−1)β*C*, *L*>*C*, α*L*>γ*C*, *c*_11_(0, 1) < 0, *c*_22_(0, 1) < 0, λ_1_(0, 1) < 0, λ_2_(0, 1) < 0, then the equilibrium point (0,1) is a stable and the only equilibrium point.

**Figure 1:**
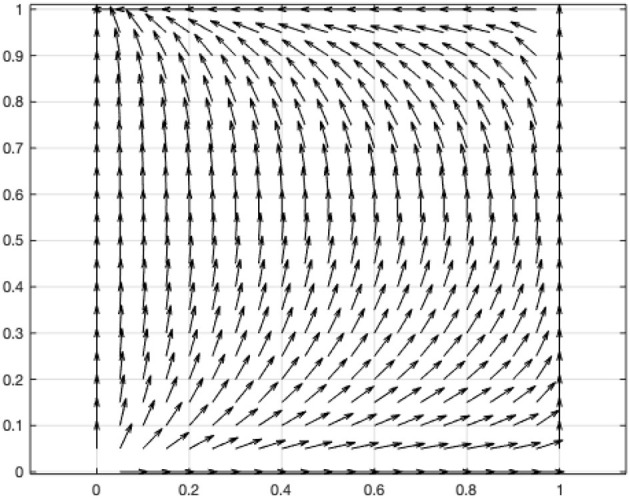
Vector diagram of two-dimensional dynamic system of the game between the government and online users.

According to this condition, the coefficients of the above two-dimensional dynamic system are assigned as *V* = 1, *L* = 0.2, *C* = 0.1, α = 1.05, β = 0.5, γ = 2, and the vector diagram of the two-dimensional dynamic system is drawn using MATLAB (Figure 1).

In the context of the pandemic and online users' comfort levels with assenting to their information being shared, the government's disclosure of the user's information will lead to a situation in which the value generated by the disclosure of the information is higher than the social costs caused by the disclosure. Therefore, the government will eventually choose to disclose the collected user information to better manage the spread of the pandemic. For the online user, when the personal interest gained by sharing personal information is less than the personal cost incurred by the government's disclosure of the user's information, the online user will choose not to share personal information with the government.

(2) When (α−1)β(*V*+*L*) < (γ−1)β*C*, *L*>*C*, α*L* < γ*C*, *c*_11_(0, 1) < 0, *c*_22_(0, 1) < 0, *c*_11_(1, 0) < 0, *c*_22_(1, 0) < 0, λ_1_(0, 1) < 0, λ_2_(0, 1) < 0, λ_1_(1, 0) < 0, λ_2_(1, 0) < 0, λ_1_(*a, b*)λ_2_(*a, b*) < 0, then the equilibrium points (0,1) and (1,0) are the stable equilibrium points, and (a,b) are saddle points.

**Figure 2:**
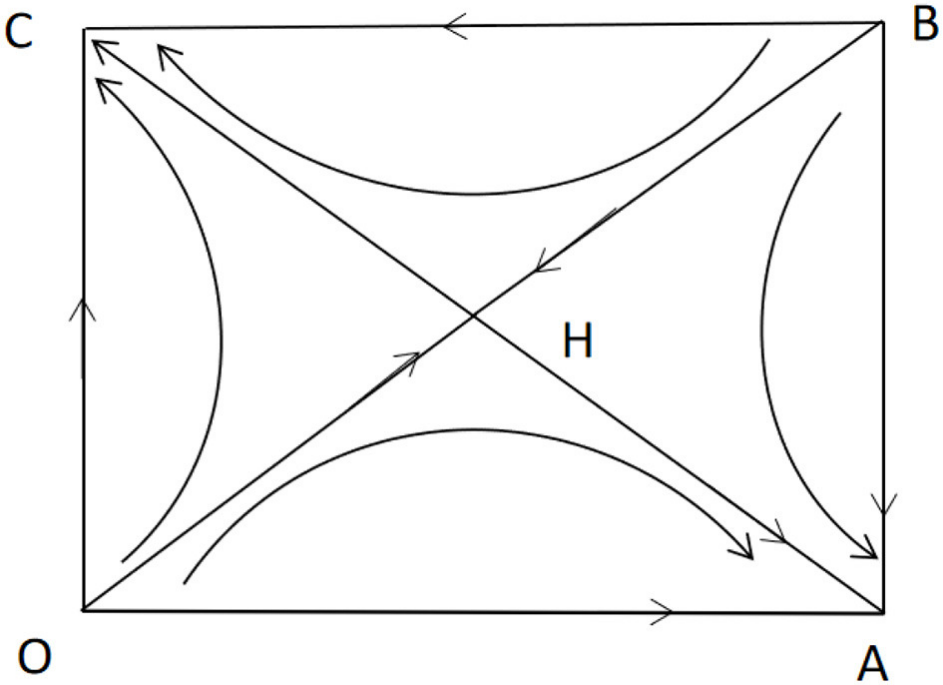
Phase diagram of two-dimensional dynamic system of game between the government and online users.

**Figure 3:**
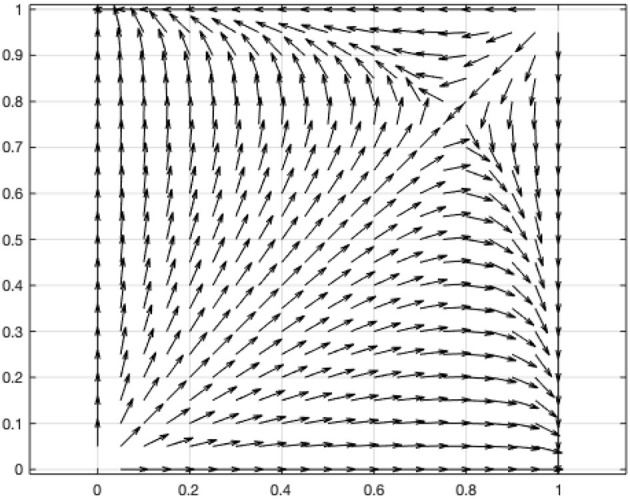
Vector diagram of two-dimensional dynamic system of game between the government and online users.

Figure 2 shows the phase diagram of government and online user decision-making. According to this condition, the coefficients of the above two-dimensional dynamic systems are assigned: *V* = 1, *L* = 0.2, *C* = 0.1, α = 1.1, β = 0.5, γ = 2.5. The vector diagram of the two-dimensional dynamic system was drawn using MATLAB (Figure 3).

The result indicates that when the user refrains from sharing personal information, the government's disclosure of the user information will lead to a situation where the value generated by the disclosure of the information is higher than the social costs associated with the disclosure (*L*>*C*). Conversely, when the user shares personal information, the government's disclosure of user information leads to a situation in which the additional value generated by disclosing the information is lower than the social costs associated with the disclosure (α*L* < γ*C*). Furthermore, when the personal interest gained by the user in sharing personal information is less than the personal cost incurred because of the government's disclosure of the user's information, the user and the government will eventually choose the strategy profiles of (not to share, to disclose) or (to share, not to disclose) and the decisions remain unchanged.

For H coordinates marked as (a,b) in the phase diagram, the probability of it converging to the equilibrium point C (0,1) is

$$\begin{array}{l} P_{C}=S_{OHBC}=\frac{1}{2}a+\frac{1}{2}\left(1-b\right)=\frac{1}{2}+\frac{L-C-\left(\alpha -1\right)V}{\left(\alpha -1\right)V-\left(\gamma -1\right)C} \end{array}$$

The probability of the two converging to the equilibrium point B (0,1) in the game is *P*_*B*_ = 1−*S*_*OHBC*_, where a higher *L* leads to a higher *P*_*C*_, and the evolutionary game of the online user and the government have a higher probability of converging to the strategy profile of (not to share, to disclose) eventually. That is, when the additional value generated by the government's disclosure of user information is higher, the government will be more inclined to disclose user information. However, simultaneously, the user will be more inclined to refrain from sharing personal information to avoid loss caused by privacy leakage.

It can be seen from $\frac{\partial P_{C}}{\partial \alpha }=\frac{V\left(\gamma C-L\right)}{{\left[\left(\alpha -1\right)V-\left(\gamma -1\right)C\right]}^{2}}$ that when γ*C*>*L*, that is, when the government's disclosure of the personal information shared by the user leads to a situation in which the additional social costs caused by privacy leakage are higher than the additional value generated by the disclosure of information, then $\frac{\partial P_{C}}{\partial \alpha }>0$. Further, if the value generated by the user's sharing of personal information for pandemic prevention and control increases, the probability of the evolutionary game of the user and the government to converge to (not to share, to disclose) will increase, whereas the probability of it converging to (to share, not to disclose) will decrease. When γ*C*< *L*, $\frac{\partial P_{C}}{\partial \alpha }<0$, and if the value generated by the user's sharing of personal information for pandemic prevention and control increases, the probability of the evolutionary game of the user and the government to converge to (not to share, to disclose) will decrease, whereas the probability of it converging to (to share, not to disclose) will increase.

**Figure 4:**
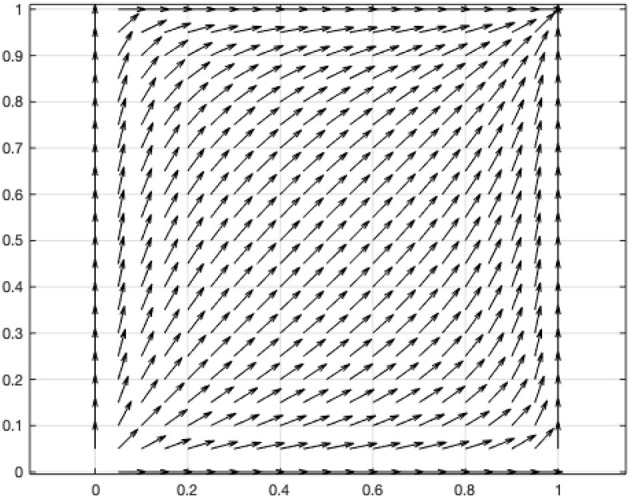
Vector diagram of two-dimensional dynamic system of the game between government and online users.

(3) When (α−1)β(*V*+*L*)>(γ−1)β*C*, α*L*>γ*C*, *c*_11_(1, 1) < 0, *c*_22_(1, 1) < 0, and λ_1_(1, 1) < 0, λ_2_(1, 1) < 0, then the equilibrium point (1,1) is a stable, and it is the only one equilibrium point. According to this condition, the coefficients of the above two-dimensional dynamic system are assigned *V* = 1, *L* = 0.2, *C* = 0.1, α = 1.1, β = 0.5, γ = 1.1. The vector diagram of the two-dimensional dynamic system is drawn using MATLAB (Figure 4).

The result indicates that in the prevention and control of the pandemic, the personal interest gained by the online user upon sharing personal information is larger than the personal cost caused by the government's disclosure of the user's information. When the government's disclosure of personal information leads to a situation in which the additional value generated by the disclosure of the information is higher than the social costs incurred by the disclosure (α*L*>γ*C*), the evolutionary game of the user and the government will eventually choose the strategy profile of (to share, disclose) and remain unchanged.

## Discussion

The previous Section “Research Design” analyzed the equilibrium results of the game of the government and online users regarding information sharing and pandemic prevention and control under different conditions. However, whether the equilibrium results represent the social optima, or in other words, the expected results of the people, remains inconclusive. In many cases, individual rationality and collective rationality diverge. When the game results of individual rationality do not lead to social optima, the Pareto improvement of the game results can be achieved through government intervention and guidance. Therefore, this section focuses on discussing the social efficiency of the equilibrium results in the games mentioned above.

(1) According to the analysis in the previous section, when (α−1)β(*V*+*L*) < (γ−1)β*C*, *L*>*C*, and α*L*>γ*C*, the final result of the evolutionary game between online users and the government is (not shared, disclosed). From the perspective of social efficiency, the game results in total social efficiency is *U*_1, 0_ = *U*_*p*, 1_+*U*_*g*, 0_ = (*V*+*L*)−*C*. When the online user and the government choose the strategies of (to share, disclose), the total social efficiency is *U*_0, 0_ = *U*_*p*, 0_+*U*_*g*, 0_ = α(*V*+*L*)−γ*C*. When the online user and government choose the strategies of (to share, not disclose), the total social efficiency is *U*_0, 1_ = *U*_*p*, 0_+*U*_*g*, 1_ = α*V*. When the online user and the government choose the strategies of (not to share, not to disclose), the total social efficiency is *U*_1, 1_ = *U*_*p*, 1_+*U*_*g*, 1_ = *V*.

From (α−1)β(*V*+*L*) < (γ−1)β*C*⇔α(*V*+*L*)−γ*C* < (*V*+*L*)−*C*, then *U*_0, 0_< *U*_1, 0_, such that when $\left(\alpha -1\right)<\frac{L-C}{V}$, then *U*_1, 0_>*U*_0, 1_>*U*_1, 1_, the game result of the online user and the government (not to share, to disclose) is the social optimum state. To elaborate, when the personal information shared by online users with the government generates a value that is too small for the government's prevention and control of the pandemic, it achieves the social optimum state when the user refrains from sharing personal information as the individual maximizes his/her personal interest. On the contrary, when the personal information shared by users is very conducive to the government's prevention and control of the pandemic, it can, in turn, promote the overall social benefits. If the user is dissuaded from sharing personal information by the loss caused by privacy leakage, and chooses not to share personal data, the social optimum cannot be achieved. In contrast, there is a conflict between individual and collective rationality. Consequently, when the value generated by online users' sharing of personal information with the government is sufficiently high enough to facilitate the government's prevention and control of the pandemic, and leads to a result in which the game results of the two players are not consistent with the social optimum, incentive policies designed for online users can be introduced to induce sharing of personal information. This is so that the game results of the two players approach the situation expected by society.

(2) According to the analysis in the previous section, when (α−1)β(*V*+*L*) < (γ−1)β*C*, *L*>*C*, and α*L* < γ*C*, the final result of the evolutionary game between online users and the government is (no sharing, disclosure) or (sharing, no disclosure). From the perspective of social efficiency, when $\left(\alpha -1\right)<\frac{L-C}{V}$, *U*_1, 0_>*U*_0, 1_, the equilibrium point (not to share, to disclose) is more optimal than (to share, not to disclose), and the equilibrium point (not to share, to disclose) is also the social optimum state. When $\left(\alpha -1\right)>\frac{L-C}{V}$, *U*_1, 0_< *U*_0, 1_, the evolutionary game of online users and the government leads to the equilibrium point (to share, not disclose) being more optimal than (not to share, to disclose), while (to share, not to disclose) is also the social optimum state. When γ*C*< *L* and when the personal information shared by online users with the government generates a value that is relatively high for the government's prevention and control of the pandemic, that is, when (α−1) is higher, then the probability of the evolutionary game of online users and the government to converge to optimal social equilibrium is also higher. Consequently, individual rationality and collective rationality do not necessarily align in this situation. However, if the government intervenes and guides users' behavioral expectations, the game results of the two players would approach the social optimum state.

(3) According to the analysis in the previous section, when (α−1)β(*V*+*L*)>(γ−1)β*C*, α*L*>γ*C*, the final result of the evolutionary game between online users and the government is (sharing, disclosure). From the perspective of social efficiency, the total value of sharing personal information with society is greater than the total cost incurred by privacy leakage. Therefore, (to share, disclose), which is the game result of the two players, is the social optimum state. In addition to this, from the perspective of individuals, the sharing of personal information results in privacy leakage of online users. However, sharing personal information also brings extremely high value in terms of the prevention and control of the pandemic. Furthermore, users can benefit greatly from this. From the government's perspective, the personal information shared by users is extremely conducive to the prevention and control of the pandemic. Additionally, it helps stabilize the economy and build a positive reputation for the government. In this case, individual rationality and collective rationality work in the same direction. Therefore, relying solely on individual rationality in the absence of any intervention, society can achieve the expected result.

## Conclusion and Implications

Big data technology has been widely used in the prevention and control of the COVID-19 pandemic. Further, the disclosure of pandemic information has improved prevention and control efficiency. However, frequent data leaks have caused conflicts between pandemic information disclosure and privacy protection. Using the evolutionary game model, this study examines the laws of behavior in the sharing and disclosure of pandemic information of the users and the government in disease prevention and control, and attempts to explain and provide solutions for the conflicts mentioned above.

### Implications for Research

During the prevention and control of the pandemic, when the government's disclosure of the personal information of online users leads to a situation in which the additional value generated is higher than the social costs caused by the disclosure, the government will inevitably choose to disclose users' personal information to the public in order to improve the efficiency of pandemic prevention and control. As to the users, when the personal interest gained by sharing personal information may not compensate for the cost of privacy leakage, they would refrain from sharing personal information. From the perspective of overall social efficiency, the game result (not to share, to disclose) is not necessarily inefficient. It is related to the value brought to the prevention and control of the pandemic by online users' sharing of personal information: on one hand, when the value of personal information shared by online users generates a social value that is higher than a certain critical value, the government's disclosure of the person's pandemic prevention information shared by online users can improve the efficiency of pandemic prevention. However, as the online users are worried about the loss caused by privacy leakage and choose not to share personal data, the game result of the two players cannot reach the social optimum, which reflects the conflicts between individual rationality and collective rationality. On the other hand, when the social value generated by online sharing of personal pandemic information is lower than a certain critical value, it will not be the best result for society if the government chooses to control the pandemic effectively at the expense of public privacy. The social optimum and the consistence of the individual rationality and collective rationality would be reached if the users choose not to share personal information while the government chooses to disclose the information.

There are two stable equilibria exist in the evolutionary game of the public and the government: (not to share, to disclose) and (to share, not to disclose). More specifically, first, when users choose not to share personal information and the government collects user information by itself, the value of the government's disclosure of the information to the society is less than the social cost. Second, when users choose to share personal information, the value of the government's disclosure of user information to society is greater than the social cost. From the perspective of social efficiency, which one of the game results is more optimal still depends on the value generated by online sharing of personal pandemic information for pandemic prevention and control: when the personal information shared by online users generates a value that is higher than a certain critical value for pandemic prevention and control, the equilibrium of (to share, not to disclose) is more optimal than (not to share, to disclose).The equilibrium of (to share, not to disclose) is also the social optimum, the government and the online users are more inclined to converge to the status of (to share, not to disclose) with the increase of the value. The results also indicates that consciously sharing the personal information is useful to improve the efficiency of the prevention and control of the pandemic and avoid the costs of privacy leakage if the government does not disclose the public's personal information. If the situation is reversed, then (not to share, to disclose) will be more optimal than (to share, not to disclose). The value generated by sharing online users' personal pandemic information is too low for pandemic prevention and control, while the risks of privacy leakage is much higher. The government and the online users are more inclined to converge to the status of (not to share, to disclose) with the decrease of the value.

When the users' personal interests from sharing personal pandemic information are greater than the personal costs incurred, and the value generated by the government's disclosure of the information is greater than the social cost it generates, the users will eventually choose to share personal pandemic information, the government will also choose to disclose the information. The result of the evolutionary game is the optimal social result, that the individual rational choice is consistent with the collective rational choice.

### Implications for Practice

Based on the above conclusions, to improve the efficiency of pandemic prevention and control, protect user privacy, and realize the unity of individual and collective rationality, the following policy suggestions are posited:

(1) Establish comprehensive rules of disclosing personal pandemic information, which offers the greatest possible protection for personal privacy rights while ensuring the government's effective use of personal information and the provision of incentives for online users to encourage sharing of personal information. In the fight against the COVID-19 pandemic, frequent information leakage incidents have exposed conflicts between information disclosure and privacy protection. The risks of privacy breach have, to some extent, discouraged the public from sharing personal information related to the pandemic. Therefore, the construction of a comprehensive disclosure mechanism of personal pandemic information, improvement in the identification of information precision, and effective technology for the concealment of privacy information can offer the greatest possible protection for personal privacy rights. For example, on March 19, 2020, the European Commission for Data Protection (EDPB) issued a statement on “personal data processing in the context of COVID-19,” which stressed that data controllers and processors must protect the personal data.

(2) Strengthen the supervision and punishment against privacy infringements related to the disclosure of online users' pandemic information. For example, data protection agencies in EU countries have stressed that the governments and enterprises should follow the privacy protection principles determined by the EU General Data Protection Regulation (GDPR) and other laws. Although the disclosure of users' information related to the pandemic can improve the efficiency of prevention and control, users pay the price as their privacy may be abused for other purposes. Therefore, for better protection of users' privacy and to improve the efficiency of pandemic prevention and control, enterprises or individuals who profit by abusing people's information related to the pandemic should be put under strict supervision and punishment. Further, their acts must be rigorously regulated to ease the worries of users. Simultaneously, they share personal information related to the pandemic and enhance their initiatives to share personal information related to the same. Therefore, as information disclosure is inevitable in preventing and controlling the spread of the virus, the best way to protect personal privacy is to strictly supervise the entities that disclose the information and hold them strictly accountable for illegal acts.

### Limitations and Further Research

Using the evolutionary game models, this study incorporates the government prevention and control decisions and online user information sharing decisions into one framework to analyze and expand existing research on the interactions between prevention and control of the pandemic and privacy protection issues. Notwithstanding, more research directions can be explored.

First, for the behavior decisions of online users, the assumption of this study focuses on whether or not to share personal information, while the precision of private information shared or disclosed by users and the government can be further incorporated into the analysis framework in the future. At the same time, the paper assumes that the distribution ratio of the value generated by the epidemic information between the government and online users remains constant. For future study, a distribution ratio that varies with the government and online users' strategies can be further explored.

Second, when constructing an evolutionary game model between government and online users, this study does not consider the interacting topology among individuals or between the individual and the government. The interacting topology between game participants will have a certain impact on the equilibrium result and the convergence process of the game (44, 45). Therefore, a consideration of the interactive topology among individuals, or between the individual and the government, will be our scope of future work.

Third, this paper describes the dynamic game process between the government and online users through replicator dynamic equations, with an awareness that the distinction between the evolutionary and the traditional games can be more fully presented by various other means. Therefore, future research should explore simulating the convergence process of the government and online users' strategies through Monte Carlo simulation, or numerical simulation, or other similar methods.

## Data Availability Statement

The original contributions presented in the study are included in the article/supplementary material, further inquiries can be directed to the corresponding author.

## Author Contributions

YX and QP conducted the modeling and data analysis and drafted of the manuscript. WX, SZ, and QP conceptualized the study, designed the experiment, and contributed to the manuscript. YX, WX, and QP contributed to the acquisition and computation of data. All authors critically revised the manuscript for important intellectual content and approved the final version.

## Funding

This work was supported by Statistical Scientific Key Research Project of China (2021LZ33) and Statistical Scientific Key Research Project of Zhejiang (21TJZZ25).

## Conflict of Interest

The authors declare that the research was conducted in the absence of any commercial or financial relationships that could be construed as a potential conflict of interest.

## Publisher's Note

All claims expressed in this article are solely those of the authors and do not necessarily represent those of their affiliated organizations, or those of the publisher, the editors and the reviewers. Any product that may be evaluated in this article, or claim that may be made by its manufacturer, is not guaranteed or endorsed by the publisher.
